# Development of an open‐pollinated genetic mapping framework to facilitate the identification of QTL in apples

**DOI:** 10.1002/tpg2.70288

**Published:** 2026-08-16

**Authors:** David Hickok, Awais Khan, Kelly Robbins

**Affiliations:** ^1^ Plant Pathology and Plant‐Microbe Biology Section, School of Integrative Plant Science Cornell University Geneva New York USA; ^2^ Plant Breeding and Genetics Section, School of Integrative Plant Science Cornell University Ithaca New York USA

## Abstract

Genetic mapping of traits in apples (*Malus domestica*), a temperate woody perennial, is challenging due to high heterozygosity, long juvenility, and extensive spatial maintenance requirements for the populations. These factors limit the effective use of traditional quantitative trait locus (QTL) mapping methods in identifying the genetic basis of traits critical for apple production, marketability, and sustainability. We explored the use of open‐pollinated (OP) genetic mapping as an alternative to conventional biparental QTL mapping and genome‐wide association studies. A QTL was simulated, and the performance of a mixed linear model (MLM) and a multi‐locus mixed model (MLMM) in QTL mapping accuracy was compared using a genotyping‐by‐sequencing dataset of seven interspecific Royal Gala × *Malus sieversii* biparental F1 populations. The simulation results show that the MLMM outperformed the MLM by accurately identifying the simulated QTL. Analysis of power indicated that a population size of 183 individuals is required to reach a power = 0.8 for a simulated major effect QTL. Mapping resolution analysis showed that a population size of 470–600 individuals, depending on local recombination rates, is necessary to achieve high resolution within the OP population. Simulations demonstrate the potential of OP‐based genetic mapping for identifying QTL in apples, reducing the logistical challenges associated with traditional QTL mapping methods. Our results show that OP‐based genetic mapping could be used to speed up the identification of novel alleles directly from diverse germplasm collections in apples.

## Core Ideas

- Biparental quantitative trait locus (QTL) mapping in apples is constrained by limited diversity, high costs, and long‐term orchard space.
- Open‐pollinated (OP)‐based mapping avoids controlled pollination, clonal propagation, and large‐scale F1 orchard requirements.
- OP‐based mapping offers an alternative to biparental mapping by leveraging existing germplasm and natural crosses.
- OP‐mapping enables QTL discovery absent full pedigree information to strategically capture broad genetic diversity.
- OP F1 populations support fast QTL detection, aiding rapid breeding against emerging pathogens.

## Plain Language Summary

Genetic mapping helps researchers locate important traits in a plant's DNA. In apples, this is usually done by manually pollinating two trees in a research orchard to create a mapping population. These orchards take a lot of time and effort to maintain, yet most of their trees are not used in mapping studies, leaving much of the orchard unused. Instead of only using two trees, this research focused on using open‐pollinated (OP) populations, where many more trees can contribute to the mapping population. This method does not require manual pollination and includes more of the orchard. To test how well OP populations could work, we simulated a trait and found locating the trait in the apple's DNA was just as effective as traditional mapping using controlled pollination. This shows that OP mapping populations are a practical and efficient alternative for genetic mapping that makes better use of the trees already growing in research orchards.

**Abbreviations**
FDR: false discovery rate
GAPIT: Genome Association and Prediction Integrated Tool
GBS: genotyping‐by‐sequencing
GRM: genomic relationship matrix
GWAS: genome‐wide association studies
K‐W: Kruskal–Wallis
LD: linkage disequilibrium
LG: linkage group
MAS: marker‐assisted selection
MLM: mixed linear model
MLMM: multi‐locus mixed model
OP: open‐pollinated
QTL: quantitative trait locus

## 1 INTRODUCTION

Apples (*Malus domestica*) are temperate woody perennials grown worldwide primarily for their nutritious fruit, making them the focus of extensive breeding efforts (Khan & Korban, 2022). Apple breeding is a lengthy and challenging process, particularly when developing cultivars that combine disease resistance with desirable fruit quality (Desnoues et al., 2018; Watts et al., 2023). The long juvenile phase of apple trees, lasting between 5 and 7 years, significantly prolongs the breeding cycle of apples (Khan & Korban, 2022). Wild apple species are often the only source of disease resistance alleles, but their introduction into commercial cultivars is hindered by linkage drag, where undesirable traits are inherited along with the desired disease resistance (Madhu et al., 2020; Stewart et al., 2023; Zhao et al., 2019). It is critical to have reliable genetic markers linked to disease resistance alleles to improve selection, reduce the linkage drag, and speed up the breeding process for developing disease‐resistant apple cultivars with good fruit quality (Desnoues et al., 2018; Watts et al., 2023).

In apples, biparental and GWASs (genome‐wide association studies) genetic mapping approaches have been used to identify the genetic basis of traits and develop markers to use in apple breeding (Khan & Korban, 2022; Ma et al., 2017). Multiple molecular marker platforms including single‐nucleotide polymorphism (SNP) arrays and genotyping‐by‐sequencing (GBS) have been developed and utilized in quantitative trait locus (QTL) mapping studies (Bianco et al., 2016; Marchini & Howie, 2010; M.‐Y. Zhang et al., 2021). While these QTL studies enabled the identification of markers closely linked to target traits, there are limitations in using these traditional QTL mapping methods in perennial, long‐juvenile, and highly heterozygous fruit crops. Biparental QTL mapping methods require controlled pollination, maintenance of populations for several years, clonal propagation, and large orchard space to achieve high‐resolution mapping (Delgado et al., 2021; Desnoues et al., 2018; Khan & Korban, 2012; Soriano et al., 2014; Tegtmeier et al., 2023). Additionally, biparental mapping is constrained by the limited genetic diversity from only two parents, which restricts its utility to mapping traits within the parents and their segregating population. However, GWAS leverages genetically diverse germplasm collections for high‐resolution mapping, but requires dense marker panels and large population sizes, making it resource‐intensive for QTL mapping (Larsen et al., 2018; Ma et al., 2017; Urrestarazu et al., 2017; Watts et al., 2023; M.‐Y. Zhang et al., 2021). Moreover, genotyping of GWAS panels in highly heterozygous species is often challenging, leading to ascertainment bias. This bias arises from incorrect SNP calls, low call rates for certain accessions, and missing data (Bianco et al., 2016).

Open‐pollinated (OP) mapping presents a promising alternative to overcome these challenges associated with traditional QTL mapping and to speed up genetic mapping in long‐juvenile and heterozygous crops and forest trees (Myles et al., 2009). Genetic mapping using OP populations, where only the maternal parent is known, can take advantage of the genetic diversity and allow for the establishment of large populations with a large number of recombinants, for high‐resolution QTL mapping (Kumar et al., 2016; Plomion et al., 1995). OP and multi‐parent populations have been used in multiple crop species and have helped to gain insight into genetic diversity, genetic characterization, and genetic mapping. In sunflower, OP populations have been used to genetically characterize various traits such as leaf morphology, head diameter, and days to maturity (Moreno et al., 2013). In Pearl Millet, a collection of selfed and OP individuals has been used to map grain iron and zinc content (Kumar et al., 2016), and in six‐row barley, multi‐parent mapping has led to the detection of 23 flowering time QTL (Hemshrot et al., 2019). Beyond nonwoody perennial species, OP genetic mapping has been successfully implemented in a variety of species, demonstrating its effectiveness in other heterozygous and long‐lived plants. For instance, in forest trees such as Japanese larch (*Larix kaempferi*) and maritime pine (*Pinus pinaster*), OP populations have enabled the identification of QTL associated with growth, adaptation, and disease resistance by leveraging large family sizes and extensive recombination events (Dong et al., 2024; Plomion et al., 1995). Genetic mapping based on OP populations could bridge the gap between GWAS and biparental QTL mapping, offering an efficient alternative for identifying marker–trait associations.

Various genetic mapping strategies have been employed for QTL mapping in both biparental populations and diversity collections (Khan & Korban, 2012). Interval mapping and composite interval mapping are traditional methods used in biparental QTL mapping, leveraging linkage information to detect QTL positions with high power (Lander & Botstein, 1989; Zeng, 1994). These methods perform well in controlled populations but are limited by the genetic diversity of the parents. For GWAS, linear models, such as the general linear model (GLM) and mixed linear model (MLM), are widely used to account for population structure and relatedness, improving the accuracy of marker–trait associations (Yu et al., 2006; Z. Zhang et al., 2010). Nonparametric approaches, such as the Kruskal–Wallis (K‐W) test, have also been employed for initial scans of marker–trait associations, and although it provides a simple and effective method for detecting associations, the K‐W test is not robust against variance heterogeneity or imbalanced genotype categories, making it less suitable for species with complex population dynamics and diverse genetic backgrounds (Konietschke et al., 2012). To improve QTL detection and power, a multi‐locus mixed model (MLMM) has shown promise in increasing the resolution and accuracy of QTL mapping by incorporating multiple loci into the model while reducing false positives (Segura et al., 2012).

The effectiveness of these mapping strategies depends on several factors, including population size, marker density, and genetic variance of the traits under investigation. Larger population sizes increase the statistical power to detect QTL and improve mapping resolution (Cockram & Mackay, 2018; Y. Xu et al., 2017). Marker density is critical in capturing recombination events, particularly in species with rapid linkage disequilibrium (LD) decay, such as apples, where high‐density SNP panels or GBS platforms are required (Khan & Korban, 2012; Myles et al., 2009). Additionally, the effectiveness of QTL mapping is influenced by the proportion of phenotypic variance explained by the QTL. QTLs with smaller effects require larger population sizes to achieve reliable detection, as larger populations provide increased statistical power and greater power in identifying QTL (Beavis, 1994; Hall et al., 2016). Accurately estimating the variance contributed by a QTL is crucial for understanding its impact on the trait and for genetic mapping (King & Long, 2017; S. Xu, 2003). Utilizing high‐density marker platforms such as SNP arrays and GBS, genetic mapping can achieve comparable QTL detection power without relying solely on traditional LM approaches (Spindel et al., 2013). Dense marker coverage enhances the resolution of association studies, enabling more precise QTL localization and marker–trait associations.

**Core Ideas**

- Biparental quantitative trait locus (QTL) mapping in apples is constrained by limited diversity, high costs, and long‐term orchard space.
- Open‐pollinated (OP)‐based mapping avoids controlled pollination, clonal propagation, and large‐scale F1 orchard requirements.
- OP‐based mapping offers an alternative to biparental mapping by leveraging existing germplasm and natural crosses.
- OP‐mapping enables QTL discovery absent full pedigree information to strategically capture broad genetic diversity.
- OP F1 populations support fast QTL detection, aiding rapid breeding against emerging pathogens.

These associations, based on their proximity to the QTL, influence the application and development of markers in the context of a breeding program. Identifying marker–trait associations tightly linked to major effect QTL is particularly important in apple breeding programs, where marker‐assisted selection (MAS) is frequently used to accelerate the introgression of disease resistance loci while minimizing linkage drag from wild germplasm (Rasool et al., 2024; Sabety et al., 2024). In breeding scenarios involving major resistance genes, such as fire blight or apple scab resistance loci, tightly linked markers can be used to screen seedlings at early developmental stages prior to field phenotyping, reducing both orchard space requirements and breeding cycle length (Rasool et al., 2024). Fine mapping of these QTLs is critical for developing reliable diagnostic markers that remain associated with the region across diverse genetic backgrounds. Increased mapping resolution within OP populations could further narrow candidate genomic intervals surrounding major effect QTL, facilitating downstream candidate gene identification and the development of markers suitable for deployment in breeding programs (López‐Girona et al., 2026; Švara et al., 2024). This advancement allows for more cost‐effective and efficient genetic mapping, particularly in highly heterozygous and complex genomes like those of woody perennials (Bianco et al., 2016).

In this study, an OP‐based genetic mapping scenario in apple was investigated using simulations to determine how effective the approach could be in identifying closely linked markers for moderate‐ to large‐effect QTL that could be deployed in a breeding program. Simulated phenotypic data for an OP genetic mapping population comprised of a collection of biparental F1 apple populations sharing a common seed parent were used to evaluate genetic models identifying marker–trait associations. Additionally, the simulation pipeline assessed the power and mapping resolution of the marker–trait associations across different population sizes. Our findings provide insights into optimizing OP‐based genetic mappings, offering an alternative method for future genetic mapping of heterozygous crops with long juvenile phases.

## 2 MATERIALS AND METHODS

### 2.1 Mapping populations and GBS SNP dataset

**FIGURE 1:**
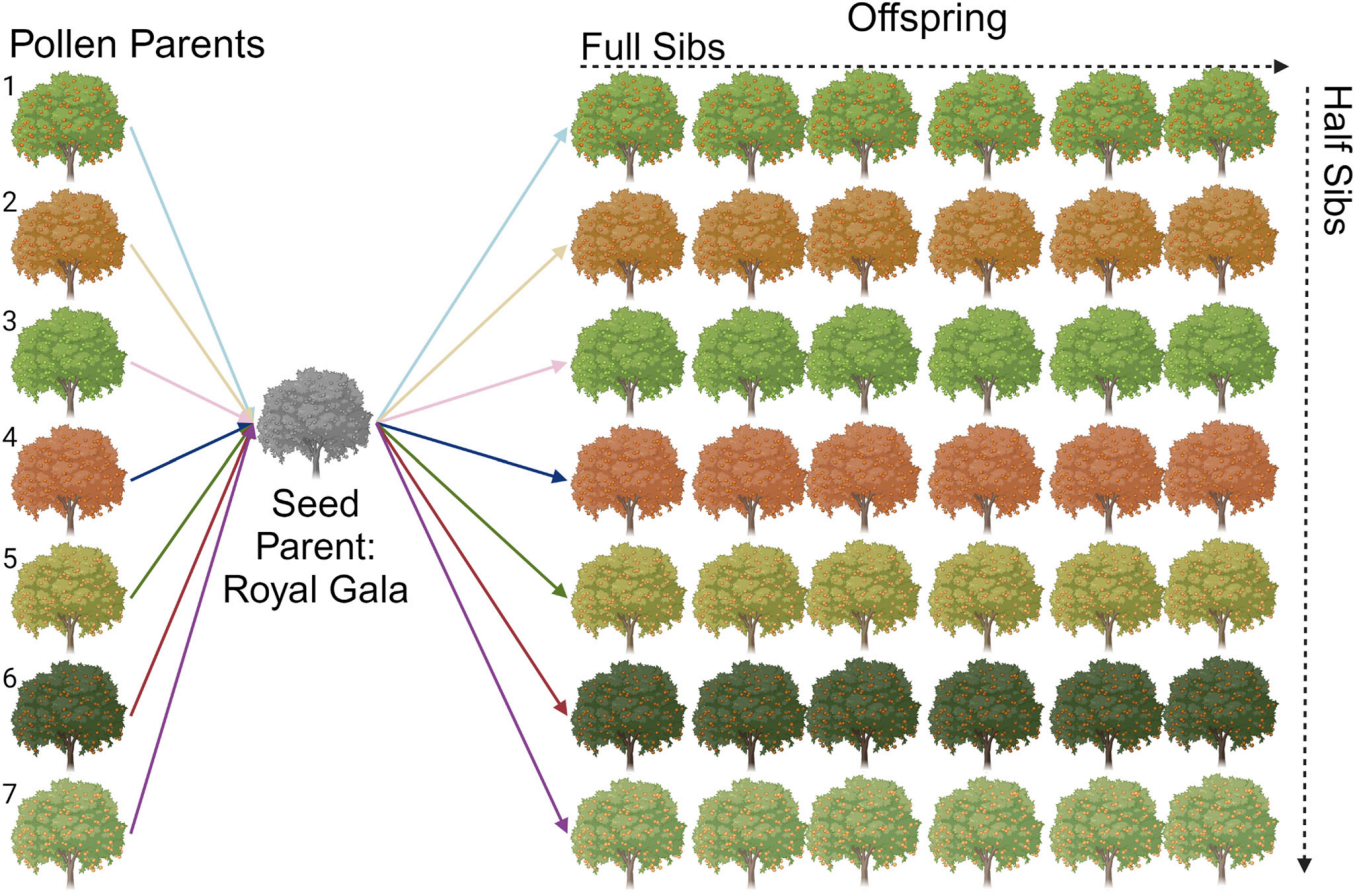
Representation of the Royal Gala (PI651008) × *M. sieversii* biparental F1 populations. The seven *M. sieversii* pollen parents shown on the left were crossed with the common Royal Gala seed parent shown in the middle. The arrows indicate the crosses and the resulting seven full‐sib populations. The F1s are aligned in rows across from the respective pollen parents, with half‐sibs aligning in columns.

A GBS dataset of seven interspecific biparental F1 populations was used to develop and evaluate an OP genetic mapping framework to identify QTL. Seven *Malus sieversii* pollen donors KAZ 95 06‐1 (PI 600497), KAZ 95 08‐0 (PI 692439), KAZ 95 10‐04F (PI 613978), KAZ 95 10‐05F (PI 600526), KAZ 95 17‐14 (PI 613959), KAZ 95 18‐07 (PI 613981), and KAZ 96 05‐04 (PI 600601) were crossed with a single maternal Royal Gala (PI651008) parent to develop this population (Figure 1). Three of these F1 populations were used and described previously in genetic mapping studies (Bai et al., 2012; Norelli et al., 2017; Tegtmeier et al., 2023). Sourcing of the paternal *M*. *sieversii* genotypes took place between 1989 and 1996 (Hokanson et al., 1997; Luby et al., 2001). These pollen parents originated from Kazakhstan and were collected and included in the *Malus* germplasm collection at the USDA–ARS Plant Genetic Resources Unit in Geneva, NY.

The GBS data for these populations were generated as part of a collaborative project between USDA–ARS, Geneva, NY, and Dalhousie University. The GBS genotyping protocol has been detailed in previous studies (Migicovsky et al., 2016, 2022; Tegtmeier et al., 2023; Thapa et al., 2021). Briefly, DNA was extracted using DNeasy 96 Plant Kits (Qiagen), and separate GBS libraries were prepared for each population following a modified version of the protocol developed by Elshire et al. (2011) utilizing ApeKI and PstI‐EcoT22I restriction enzymes. Sequencing of libraries was performed on an Illumina Hi‐Seq 2000 platform, with 96 samples per lane, at Cornell University (Ithaca, NY, USA). The resulting 100‐bp single‐end reads were filtered, and SNPs were called against the *M. domestica* GDDH13 v1.1 reference genome (Daccord et al., 2017) as described by Migicovsky et al. (2021). The progeny are all half‐siblings, allowing the data to be treated as OP when analyzed in aggregate and ignoring pollen donor identity.

### 2.2 Simulation of phenotypes and QTL

Marker associations were identified using simulated phenotypes and the Royal Gala (PI651008) × *M*. *sieversii* GBS dataset. The following model representing the sampled phenotypes was used:

$$y_{ijs}=q_{sj}+g_{si}+\varepsilon_{ijs}$$

In this equation, $y_{ijs}$​ represents the phenotypic value of the ith plant, where $q_{sj}$ is the effect of QTL genotype j from simulation scenario s; $g_{si}$ is the genetic background effect of the ith plant (polygenetic background effect for the trait of interest) sampled using the parameters for simulation scenario s; and $\varepsilon_{ijs}$ is the random residual error. The QTL effect $q_{sj}$ was simulated to explain either 0.5 or 0.8 of the total genetic variance dependent on the simulation scenario s. The term q was simulated as $q_{s}∼\mathrm{MVN}\left(0,I\sigma_{q_{s}}^{2}\right)$, where $q_{s}$​ is a vector with two values representing the effect and non‐effect alleles. The genetic background effect was simulated as $g_{s}$∼MVN(0, $G\sigma_{g_{s}}^{2}$), where $g_{s}$ is a vector of genetic background effects with a length equal to the sample size of simulation scenario s, MVN is multivariate normal; G is the genomic relationship matrix (GRM) calculated using the VanRaden method (VanRaden, 2008), and $\sigma_{g_{s}}^{2}$​ is the scalar representing the polygenic variance, which would explain 0.5 or 0.2 of the total genetic variance depending on the magnitude of the genetic variance explained by the QTL effect, such that the total genetic variation remained constant across all simulation scenarios. The residual term $\varepsilon_{ijs}$ captures unexplained variation and was sampled as ε∼MVN(0, $I\sigma_{e_{s}}^{2}$), where I is the identity matrix and $\sigma_{e_{s}}^{2}$​ is the residual variance contributing to the phenotypic value y, with the value of $\sigma_{e_{s}}^{2}$ set to achieve the desired heritability of simulation scenario s. All experiments were simulated as unreplicated.

The trait was simulated such that the allelic state of the Royal Gala maternal parent was heterozygous at the locus (A/a), while all paternal *M. sieversii* parents were homozygous (a/a) at the locus. This configuration ensured the segregation of the allele unique to the seed parent within the F1 population, with inheritance of the QTL exclusively derived from the allelic state of the seed parent, Royal Gala. By doing so, each progeny plant inherited either the allele unique to the seed parent allele or the non‐effect allele from the seed parent, allowing for segregation between effect and non‐effect phenotypes based on the presence or absence of the seed parent allele.

All simulation scenarios included a single major or moderate effect QTL. QTL effects were simulated under an additive genetic model, with no dominance effects included. QTL positions were fixed within predefined LD regions, allowing consistent comparison across simulations. No explicit minor allele frequency thresholds were imposed during simulation, and observed allele frequencies result from segregation in the F1 population. Residual variance was held constant within each simulation scenario, while genetic variance was varied to achieve the two heritabilities. Although only a single major QTL was explicitly simulated, the inclusion of polygenic background effects and residual variance results in phenotypes influenced by many small‐effect loci, approximating a complex genetic architecture rather than a purely single‐locus trait. The distributions of simulated phenotype values are shown across all repeated simulation scenarios and population sizes for linkage group (LG) 1 and LG 7 (Figure S1).

#### 2.2.1 Simulation parameters

**FIGURE 2:**
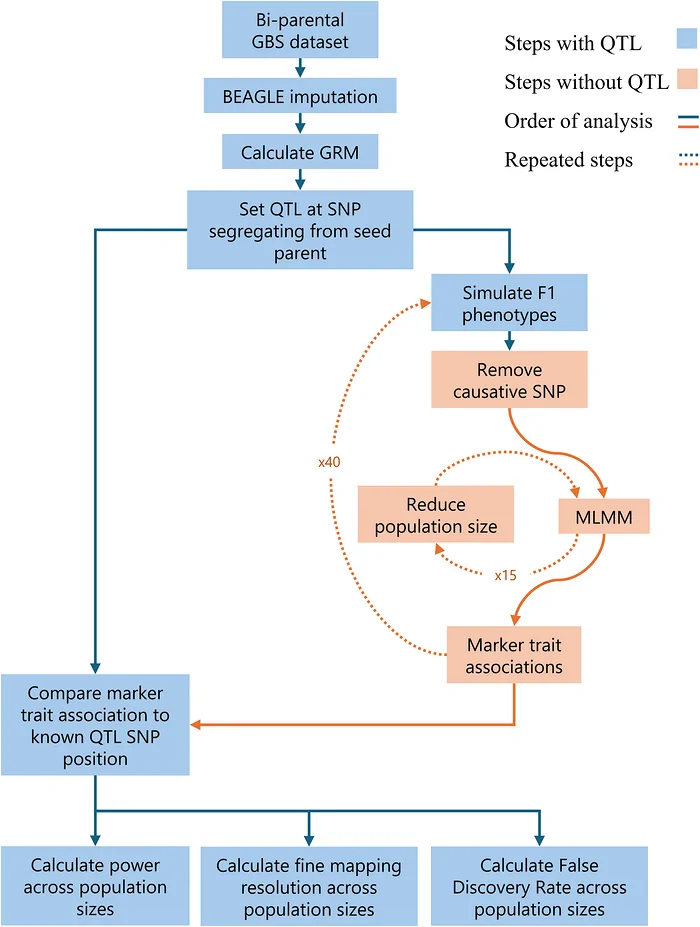
Workflow used to calculate power, false discovery rate (FDR), and determine resolution of marker–trait associations. Blue arrows indicate the sequence of steps involved in simulating phenotypes and analysis of marker–trait associations including the quantitative trait locus (QTL). Orange arrows indicate the direction of simulation and analysis where the QTL is masked and do not include the simulated QTL. Dashed lines indicate steps that are repeated. Orange boxes indicate steps excluding the QTL. Blue boxes indicate steps that include the QTL. First, the biparental genotyping‐by‐sequencing (GBS) dataset is used to calculate a genomic relationship matrix (GRM). Next, the multi‐locus mixed model (MLMM) is run 15 times with a reduced population size each run. Phenotypes are simulated 40 times for the population. The process of obtaining marker–trait associations from these simulated phenotypes is repeated 40 times, with new phenotypes being simulated for each repetition. Marker associations are compared to the position of the simulated QTL to calculate power, FDR, and fine‐mapping resolution. SNP, single‐nucleotide polymorphism.

To evaluate the effect of population size on QTL detection, a multi‐step procedure was used. First, the GBS dataset was imputed using BEAGLE (Browning et al., 2018), and the biparental GBS dataset was used to calculate a GRM for all individuals. Next, the MLMM was run 15 times, each time on a reduced population size. For each of these population sizes, phenotypes were simulated under the predefined QTL parameters (Table 1). The simulated phenotypes were then analyzed with the MLMM to obtain marker–trait associations. We repeated this process 40 times for each reduced population size, generating new simulated phenotypes in each repetition. Finally, the identified marker–trait associations were compared to the location of the simulated QTL to calculate both power, false discovery rate (FDR), and fine‐mapping resolution (Figure 2).

**TABLE 1:** Simulation parameters for all scenarios for model comparison and power, false discovery rate (FDR), and resolution comparisons. Scenarios 1 and 2 were located on linkage group (LG) 3 with a heritability of 0.8 and genetic variance of 0.8, while Scenario 6 was located on LG 7 with a heritability of 0.89 and genetic variance of 0.5, based on parameters for a previously identified fire blight resistance quantitative trait locus (QTL), MSV_FB7. For Scenarios 4–7, the heritability was set to 0.5, with corresponding genetic variances of 0.5 or 0.8, respectively. Simulations were conducted on two LGs (LG 7 and LG 1) categorized with higher (linkage disequilibrium [LD] = 0.3) or lower (LD = 0.03).

Simulation parameters: MLM and MLMM					
Scenario	Heritability	Genetic variance	Linkage group	SNP	Model
1	0.80	0.80	LG 3	SLG3_PHT_2552347	MLM
2	0.80	0.80	LG 3	SLG3_PHT_2552347	MLMM
3	0.89	0.50	LG 7	SLG7_PHT_27422647	MLMM

Simulation parameters: Power, FDR, Resolution						
Scenario	Heritability	Genetic variance	Linkage group	LD level	SNP	Model
4	0.50	0.50	LG 7	0.30	SLG7_PHT_27636570	MLMM
5	0.50	0.80	LG 7	0.30	SLG7_PHT_27636570	MLMM
6	0.50	0.50	LG 1	0.03	SLG1_PHT_18796150	MLMM
7	0.50	0.80	LG 1	0.03	SLG1_PHT_18796150	MLMM

Abbreviations: MLM, mixed linear model; MLMM, multi‐locus mixed model; SNP, single‐nucleotide polymorphism.

To assess how the proportion of genetic variance explained by a QTL, we simulated phenotypes from the GBS dataset using two values of $V_{G}$ (0.5 and 0.8). In addition, we incorporated a heritability of 0.5 to represent scenarios where disease resistance traits in apple can exhibit moderate heritability, as reported for fire blight (Kostick et al., 2021), apple scab (Bus et al., 2002), and powdery mildew (Sestras et al., 2011). Accordingly, the simulated QTL in simulation scenarios (4–7) approximate the moderate‐heritability levels that have been reported for some disease resistance traits in apple (Table 1).

### 2.3 Imputation of genotypic data

Inconsistent and missing genotype calls are a feature of lower coverage GBS datasets, where limited read depth and allelic dropout frequently lead to heterozygote undercalling and inconsistencies, even in controlled crosses (Glaubitz et al., 2014). In highly heterozygous, outcrossing perennial species such as apple, accurate genotype calling can be more challenging despite pedigree correctness (Antanaviciute et al., 2012; Bianco et al., 2014).

To ensure the correctness of genotype calls in the F1 populations, we first identified and removed impossible allelic states based on parental genotypes. Specifically, the maternal parent (*Royal Gala*) was heterozygous at the locus of interest, while all paternal parents (*M. sieversii*) were homozygous recessive and did not carry the desired allele. Consequently, any progeny genotype that did not conform to these parental configurations (e.g., A/A when the paternal parent is a/a) was deemed impossible and set to missing “NA.” This quality control step was done to minimize genotyping error in downstream analysis conducted under OP assumptions to ensure consistent genotypes throughout the population and minimize genotyping error in downstream analysis conducted under OP assumptions. To address the resulting missing data, we employed BEAGLE for genotype imputation. BEAGLE was utilized without incorporating parental genotype information, treating the dataset as if parentage were unknown. This approach leveraged the LD patterns and haplotype structures within the population to accurately infer the missing genotypes agnostic to potential parentage. The filtered GBS dataset was formatted according to BEAGLE's requirements and imputed using standard parameter settings, including default iteration counts and effective population size estimates. Phenotypes were simulated based on genotype calls after removal of impossible allelic states and imputation, ensuring genotyping error would have no impact on assessments of the performance of OP mapping.

### 2.4 QTL localization

To investigate the ability to fine‐map QTL in OP populations of apples, QTLs were simulated within genomic regions defined by differing levels of LD. Two (25 kb) regions, one with low average LD (0.03) across the region and one with higher LD (0.30) across the region, were chosen, representing higher and lower recombination regions. The workflow of the simulation is represented in Figure 2.

### 2.5 Model selection for genetic mapping

Evaluation of different mapping approaches was performed to determine the effectiveness of two different MLMs: the MLM and the MLMM. The MLM accounts for population structure by incorporating a kinship matrix, thereby controlling for relatedness among individuals. In contrast, the MLMM extends the MLM by additionally accounting for confounding background genetic effects through the inclusion of multiple genetic markers as fixed covariates using a stepwise regression approach (Segura et al., 2012). MLMM has been used to identify large‐effect QTL to clarify complex association signals (Lipka et al., 2013). In the case of apples and the use of OP populations, there will be multiple pollen donors leading to multiple pollen parents in the F1 population; accurate marker–trait associations require a model capable of accounting for the heterozygosity and population structure common in these populations (McClure et al., 2018; Minamikawa et al., 2024). MLMM accounts for these factors by iteratively incorporating significant markers as fixed‐effect cofactors into a mixed model framework that controls for relatedness and structure and has been used to identify major loci underlying complex traits in structured plant populations (Lipka et al., 2013; Segura et al., 2012). Both models were implemented using the Genome Association and Prediction Integrated Tool (GAPIT v3.0) within the R (version 4.2.2) environment (Lipka et al., 2012). All genotypic and simulated phenotypic data were formatted according to GAPIT standards. Comparison of MLM and MLMM for a single QTL with heritability of 0.8 and percent genotypic variation explained of 0.8, similar to that of MR5, a fire blight resistance gene located on LG 3 (Peil et al., 2007). Additionally, the simulation parameters were informed by findings from previous research on the genetic architecture of the biparental subpopulation M4591, specifically focusing on *MSV_FB7*. *MSV_FB7* is a fire blight resistance locus with an effect size of 0.5 and a heritability of 0.89, located on LG 7 (Tegtmeier et al., 2023).

### 2.6 Linkage mapping and OP mapping comparison

The heritability, variation explained, and location of the QTL were set similar to the *MSV_FB7* fire blight resistance QTL (Tegtmeier et al., 2023) for simulations and to compare an OP‐based genetic mapping method to a biparental QTL mapping method. Heritability was set to 0.89, and variance explained by the locus was set to 0.5. Evaluation of different mapping approaches was performed to determine the effectiveness of the MLM and the MLMM. Based on initial comparisons between the MLM and MLMM (Scenarios 1–3), we selected the MLMM as our primary mapping approach for subsequent simulations. In Scenarios 1 and 2, we modeled a major effect QTL (​ $V_{G}$ = 0.8 and *h* ^2^ = 0.8) on LG 3, reflecting parameters similar to *MR5*, a known fire blight resistance locus (Peil et al., 2007). Scenario 6 further examined a moderate effect QTL ($V_{G}$ = 0.5 and h^2^ = 0.89) on LG 7, reflecting parameters similar to *MSV_FB7*, another known fire blight resistance locus (Tegtmeier et al., 2023). Having identified the MLMM as superior in these reference cases, we next evaluated how this method performs over multiple simulated Scenarios 4–7, where the heritability was set to 0.5 and genetic variance was set to either 0.5 or 0.8 (Table 1). These lower heritabilities and varying LD levels (LG 1 vs. LG 7) provide a broader assessment of OP‐based genetic mapping in apple, as moderate effect loci are common in breeding programs and the LD landscape will be different depending on the genetic architecture surrounding the QTL.

### 2.7 Effect of population size on the OP‐based genetic mapping

**FIGURE 3:**
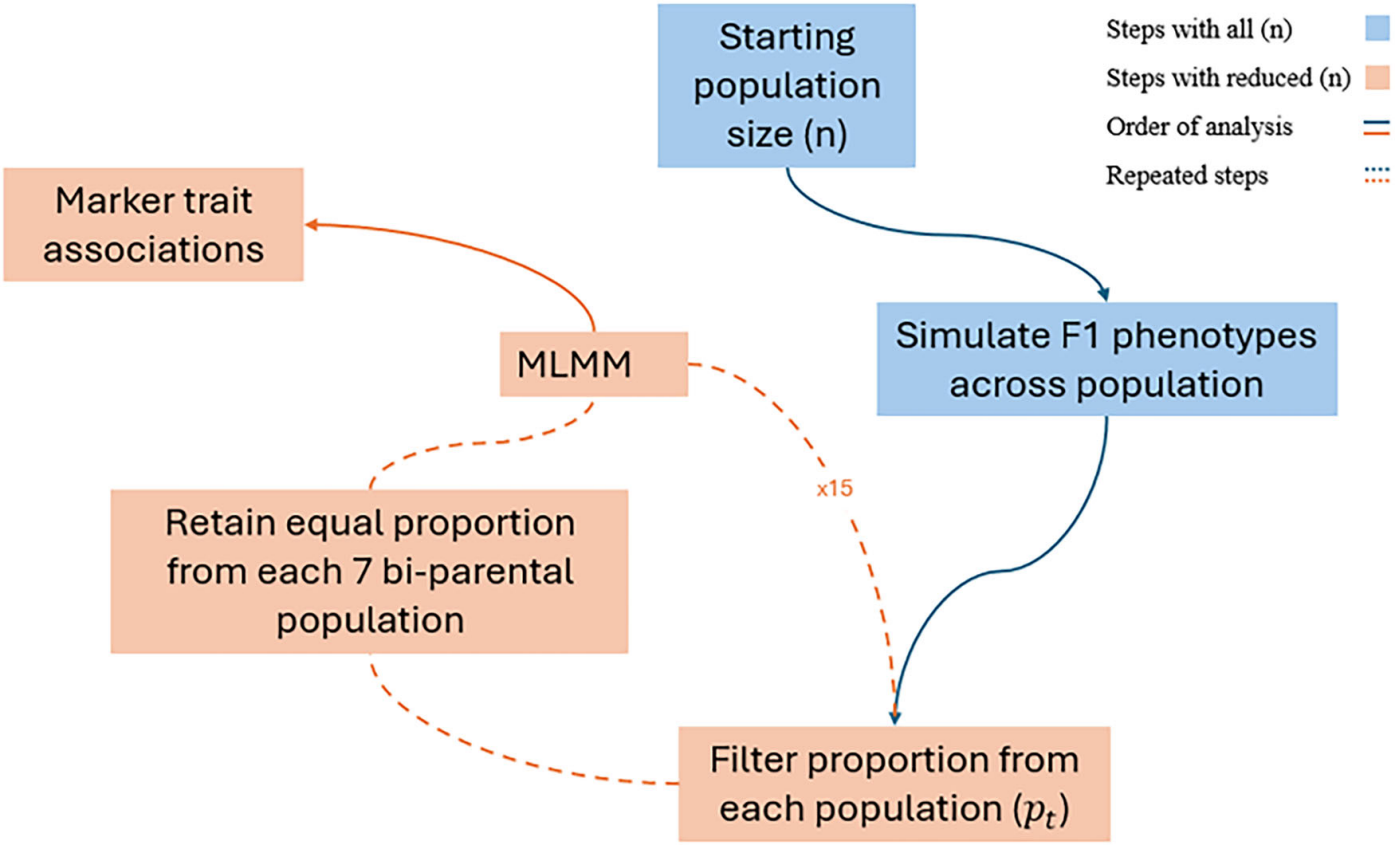
Workflow used for the population‐size reduction analysis to evaluate the effect of sample size on open‐pollinated (OP)‐based genetic mapping performance. Blue boxes and arrows indicate steps performed using the full population size (*n*), while orange boxes and arrows indicate steps performed after reducing population size (*n*). The starting population was first used to simulate F1 phenotypes across the full dataset. For each reduction level $\left(p_{t}\right)$, an equal proportion of individuals was randomly removed from each of the seven biparental subpopulations so that their relative representation remained constant. The reduced populations were then analyzed using the multi‐locus mixed model (MLMM) to generate marker–trait associations. Dashed arrows indicate repeated steps, where the reduction and analysis process was performed across 15 population sizes.

Individuals were filtered out of the dataset to determine the effect of population size on the OP‐based genetic mapping framework by calculating the power of correctly identifying significant SNPs using the MLMM. The population was filtered such that the proportion of individuals from each biparental population remained constant. This was done by removing an equal percentage of individuals from each of the biparental populations before running the MLMM with the simulated phenotypes (Figure 3). To maintain an equal proportion of each of the seven biparental populations within each simulated trial a percent of each biparental population was randomly excluded from the dataset for every round of simulations as follows: $N_{t}=\sum_{i=1}^{7}N_{i,t}=\left(1-p_{t}\right)\sum_{i=1}^{7}N_{i},t=\left(1,\text{…},15\right),$where $N_{t}$ = the trial population post reduction, $N_{i}$= the number of individuals in the subpopulation, and $p_{t}$= the proportion removed each trial.

### 2.8 Power analysis and mapping resolution

Statistical power was evaluated to determine the probability of correctly identifying a true QTL under the conditions of the simulations. Power was calculated for each population size as the proportion of simulation iterations in which at least one SNP within a specified range of the true causative QTL was identified as significant. Although LD in apple decays rapidly, often within hundreds of base pairs (Migicovsky et al., 2016), the marker density of GBS datasets is relatively sparse, resulting in large physical distances between informative SNPs. As a result, significant marker–trait associations may occur at substantial distances from the underlying causal variant. A 2 Mb window therefore provides a conservative threshold for capturing true QTL signals while accounting for the combined effects of rapid LD decay and limited marker density. To further evaluate the sensitivity of the framework for identifying candidate genes, a narrower 500 kb window was also examined. This tighter threshold provides a more biologically conservative criterion for declaring a marker as linked to the causal QTL and was selected to better reflect the comparatively rapid LD decay observed in apple populations. Comparing performance across the 500 kb and 2 Mb windows allows assessment of whether the reported power and FDR estimates depend on the choice of window size, while also testing whether the framework can localize QTL to intervals sufficiently narrow to support downstream candidate gene identification, fine mapping, and MAS. The power analysis involved simulating phenotypes with known effects and performing marker–trait associations using the MLMM. SNPs were considered significant if their *p*‐values fell below the Bonferroni‐corrected threshold of 0.05. Power was then calculated as the ratio of simulation runs where the QTL was detected to the total number of simulation runs. The equation used is given below:

$$\mathrm{Power}=\frac{\sum_{i\;=\;1}^{N}I\left(d_{i}\leq w\right)}{N}$$

where N is the total number (40) of simulations for each population size; $d_{i}$ is the distance between the closest significant SNP identified in iteration i and the true causative QTL; w is a window size of either 500 kb or 2 Mb; and $I(d_{i}\leq \;w)$ is an indicator function that equals 1 if at least one SNP within a 2 Mb or 500 kb window range of the simulated QTL was identified as significant in iteration i, and 0 if there was no SNP within the window of the simulated QTL. This analysis was conducted across different scenarios that varied in genetic variance, LD levels, and population sizes. By evaluating power under these varying conditions, we were able to determine the effectiveness of our OP‐based genetic mapping framework in reliably identifying true QTLs. The resulting power estimates provided a comprehensive understanding of the framework's capability to detect genetic associations under diverse genetic architectures and population structures.

Mapping resolution was evaluated using three complementary distance metrics calculated relative to the simulated causal QTL: the distance between the simulated QTL and the closest significant SNP, the average distance across all significant SNPs, and the distance to the furthest significant SNP identified on the chromosome containing the simulated QTL. Significant associations occurring beyond the power analysis windows of 500 kb and 2 Mb were included in the calculation, and lower distance values indicate greater mapping precision and improved QTL localization within the chromosome. For each simulation iteration, the physical distance in base pairs between the causal QTL position and each significant SNP identified by the MLMM was calculated. The minimum distance represented the closest significant SNP distance for each simulation, the average distance across all significant SNPs represented the mean significant SNP distance, and the maximum distance represented the furthest significant SNP distance for each simulation to the simulated QTL. Mean values for each metric were then calculated across the 40 simulation iterations for each population size and scenario.

$$\left|\mathrm{SN}P_{C}-\mathrm{SN}P_{\mathrm{SIG}}\right|=\mathrm{Resolution}$$

where $\mathrm{SN}P_{C}$ is the causative SNP and $\mathrm{SN}P_{\mathrm{SIG}}$ is either the maximum, minimum, or mean of the SNPs in base pairs. For each simulation, the distance between SNP_C_ and the maximum, minimum, or mean SNP_SIG_ was calculated across all simulation iterations to determine the mapping resolution.

### 2.9 False discovery rate

The FDR was calculated to determine the proportion of false positive SNPs among those identified as significantly associated with the simulated QTL. The FDR was determined using the formula:

$$\mathrm{FDR}=\frac{N_{\mathrm{FP}}}{N_{\mathrm{SIG}}}$$

where *N*_FP_ represents the number of false positive SNPs, those identified as significant but located outside a 2 Mb window surrounding the simulated QTL, and *N*_SIG_ denotes the total number of SNPs identified as significant.

## 3 RESULTS

### 3.1 Imputation

Across the 915 F1 individuals and 34,280 genotyped sites, we identified a total of 2,603,916 allelic states (8.3% of all calls) that were biologically impossible given the parental configurations described above. These calls were subsequently set to missing “NA,” which combined with the existing 1,927,671 missing sites, yielded 4,531,587 missing data points in total. This quantity accounted for approximately 14.45% of the 31,361,200 genotype calls in the dataset.

### 3.2 Model comparison between MLM and MLMM

**FIGURE 4:**
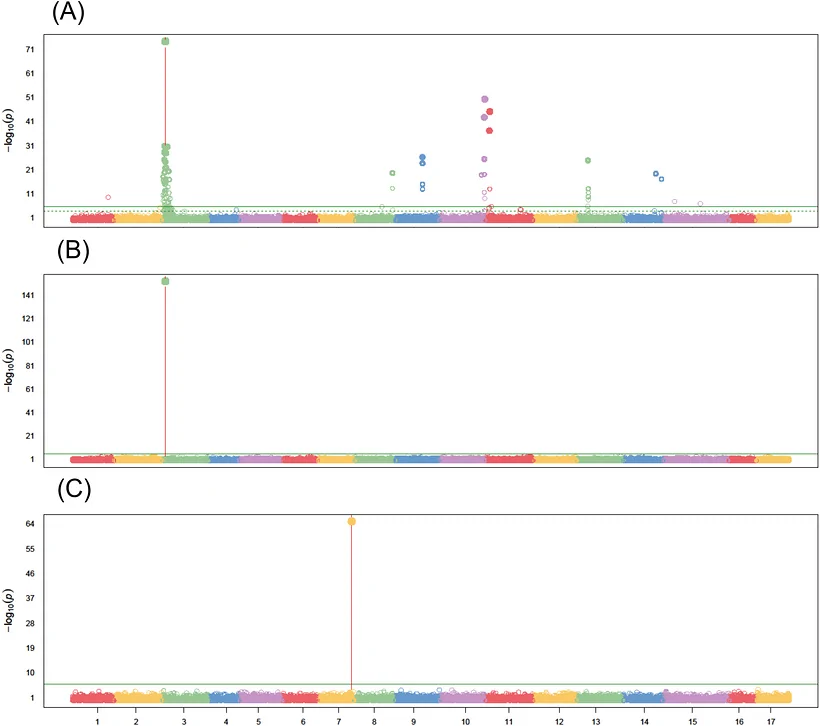
(A and B) Manhattan plots comparing simulation Scenarios 1 and 2 marker–trait associations using the same simulated quantitative trait locus (QTL) and phenotypes using two different models, mixed linear model (MLM) and multi‐locus mixed model (MLMM). MLM was used for marker–trait associations in simulation Scenario 1 in panel A, and MLMM was used for marker–trait associations in simulation Scenario 2 in panel B using the dataset from the Royal Gala (PI651008) × *M. sieversii* biparental F1 populations. The dashed line is the genome‐wide significance threshold, and the solid line is the Bonferroni‐corrected threshold of 0.05. The *x*‐axis shows the chromosome number, and the *y*‐axis is −log10(*p*) value. single‐nucleotide polymorphisms (SNPs) are represented by different colored points, with the colors indicating the linkage group (LG) the variant is located in. A red vertical line indicates the location of the simulated QTL. The SNPs detected by the MLM were on nine different LGs, and the SNP detected by the MLMM was the same as the one selected for the simulated QTL SLG3_PHT_2552347. (C) Manhattan plot of marker–trait association results for simulation Scenario 3 with the same *h* ^2^, V_G_, and location as a previously identified fire blight resistance QTL on LG07, MSV_FB7. The Manhattan plot for the simulated QTL shows the linked SNP SLG7 PHT 27422647 within the previously identified MSV_FB7 QTL region (Tegtmeier et al., 2023) between markers SLG7_PHT_22699693 and SLG7_PHT_27725770.

A simulated QTL with parameters identical to the previously reported fire blight resistance QTL, *MSV_FB7*, that is, $h^{2}=0.89$, $V_{G}=0.5$, which segregates in the biparental population GMAL 4591, was successfully identified using the MLMM on LG 7 (Figure 4C). The associated SNP, SLG7_PHT_27422647, showed a highly significant association (*p*‐value = 7.95e‐146) with the trait and this SNP falls within the previously identified QTL region for *MSV_FB7*, spanning from markers SLG7_PHT_22699693 to SLG7_PHT_27725770. The comparison between the MLM and the MLMM for a single QTL was done using simulation Scenarios 1–3 (Table 1), highlighting distinct patterns in the association profiles of each model.

The MLM identified nine SNP marker peaks across nine different LGs, suggesting erroneous associations with the simulated QTL. In contrast, the MLMM isolated a single, strong association precisely at the simulated QTL on LG 3, reducing the unrelated associations found with the MLM. Figure 4A demonstrates that the MLM's associations were dispersed across multiple LGs, potentially due to population structure or unaccounted background genetics, whereas Figure 4B shows the MLMM pinpointing only the intended QTL region. These results illustrate that the MLMM approach provides more targeted associations by controlling for confounding effects. Consequently, the MLMM proved more effective in minimizing spurious associations, enhancing the detection of the simulated QTL. This reduction in extraneous peaks validated MLMM's suitability for the genetic structure of OP apple populations and led to its selection for all subsequent simulations.

### 3.3 Power analysis, false discovery rate, and fine‐mapping resolution under different levels of $V_{G}$

**FIGURE 5:**
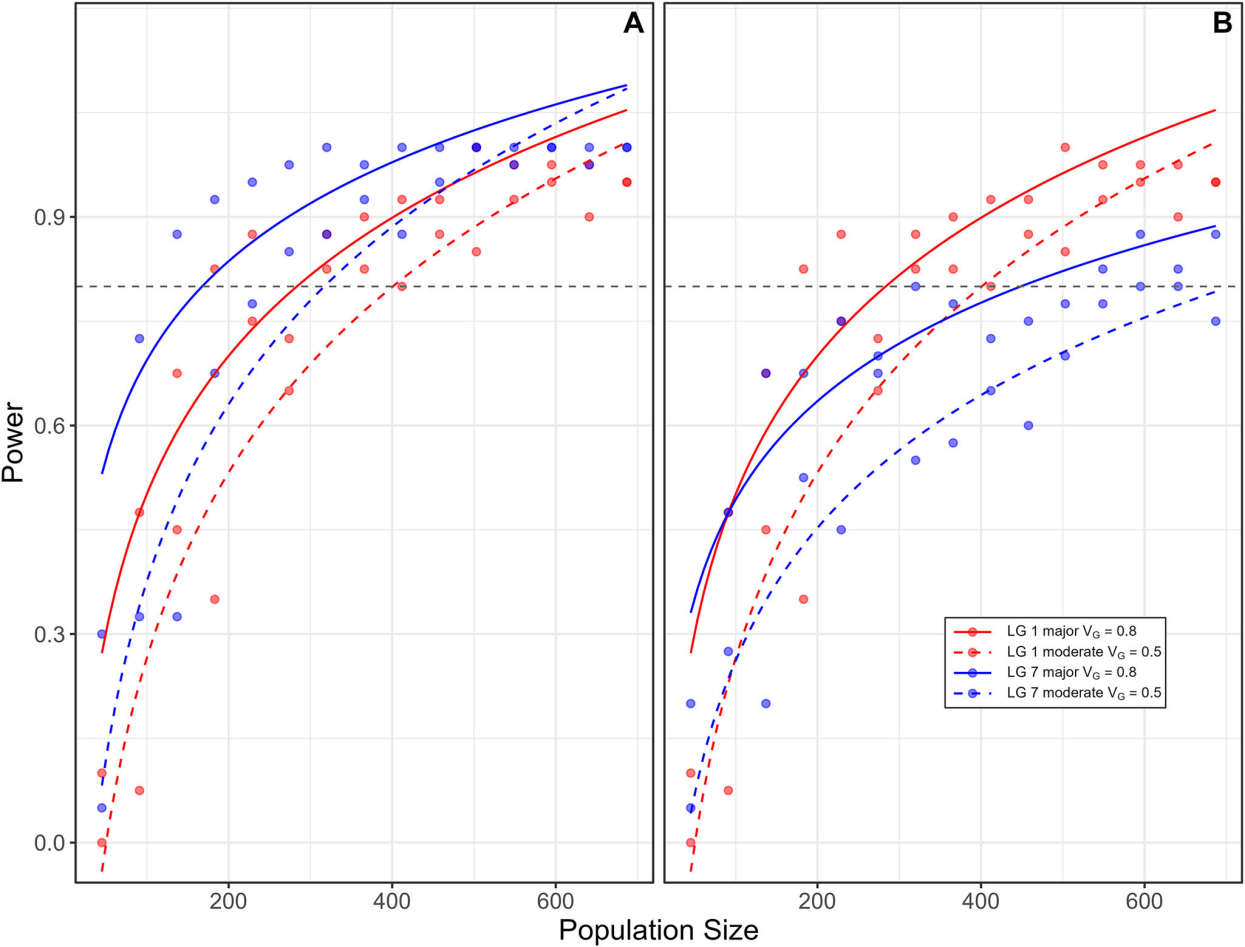
Power analysis of marker–trait associations for simulated quantitative trait locus (QTL) on linkage group (LG) 1 and LG 7 using the multi‐locus mixed model (MLMM) across 15 population sizes. Statistical power is shown as the proportion of 40 simulation replicates in which at least one significant single‐nucleotide polymorphism (SNP) was identified within a specified window surrounding the simulated QTL. Population size is shown on the *x*‐axis and statistical power on the *y*‐axis. (A) Power calculated using a 2 Mb window surrounding the causal QTL. (B) Power calculated using a 500 kb detection window surrounding the causal QTL. Red lines indicate simulations on LG 1 and blue lines indicate simulations on LG 7. Major effect QTL simulations (*V*_G_ = 0.8) are represented by solid lines, while moderate effect QTL simulations (*V*_G_ = 0.5) are represented by dashed lines. Points represent observed power estimates across 40 simulation replicates and lines represent fitted log‐linear trends. The horizontal dashed line indicates the statistical power threshold of 0.8.

Over different proportions of genetic variations explained by the simulated QTL ($V_{G}=0.5$ and $V_{G}=0.8$) on both LG 1 and LG 7, it was observed that the number of individuals in the population required to achieve a statistical power of 0.8 within either a 500 kb window or 2 Mb window around the simulated QTL decreased with a higher $V_{G}$ and lower recombination rate. For the simulated QTL on LG 1, the major effect QTL with $V_{G}=0.8$ required a population size of 183 individuals to reach the power threshold 0.8 within both the 500 kb and 2 Mb windows (Figure 5, Table S1). In contrast, the moderate effect QTL on LG 1 with $V_{G}=0.5$ required a larger population size of 320 individuals to achieve the same power threshold within both the 500 kb and 2 Mb windows. For the simulated QTL on LG 7, the major effect QTL with $V_{G}=0.8$ required a population size of 91 individuals within the 2 Mb window and 320 individuals within the 500 kb window to reach a power of 0.8, while the moderate effect QTL on LG 7 required a population size of 91 individuals within the 2 Mb window and 595 individuals within the 500 kb window to achieve the same power (Figure 5, Table S1).

**FIGURE 6:**
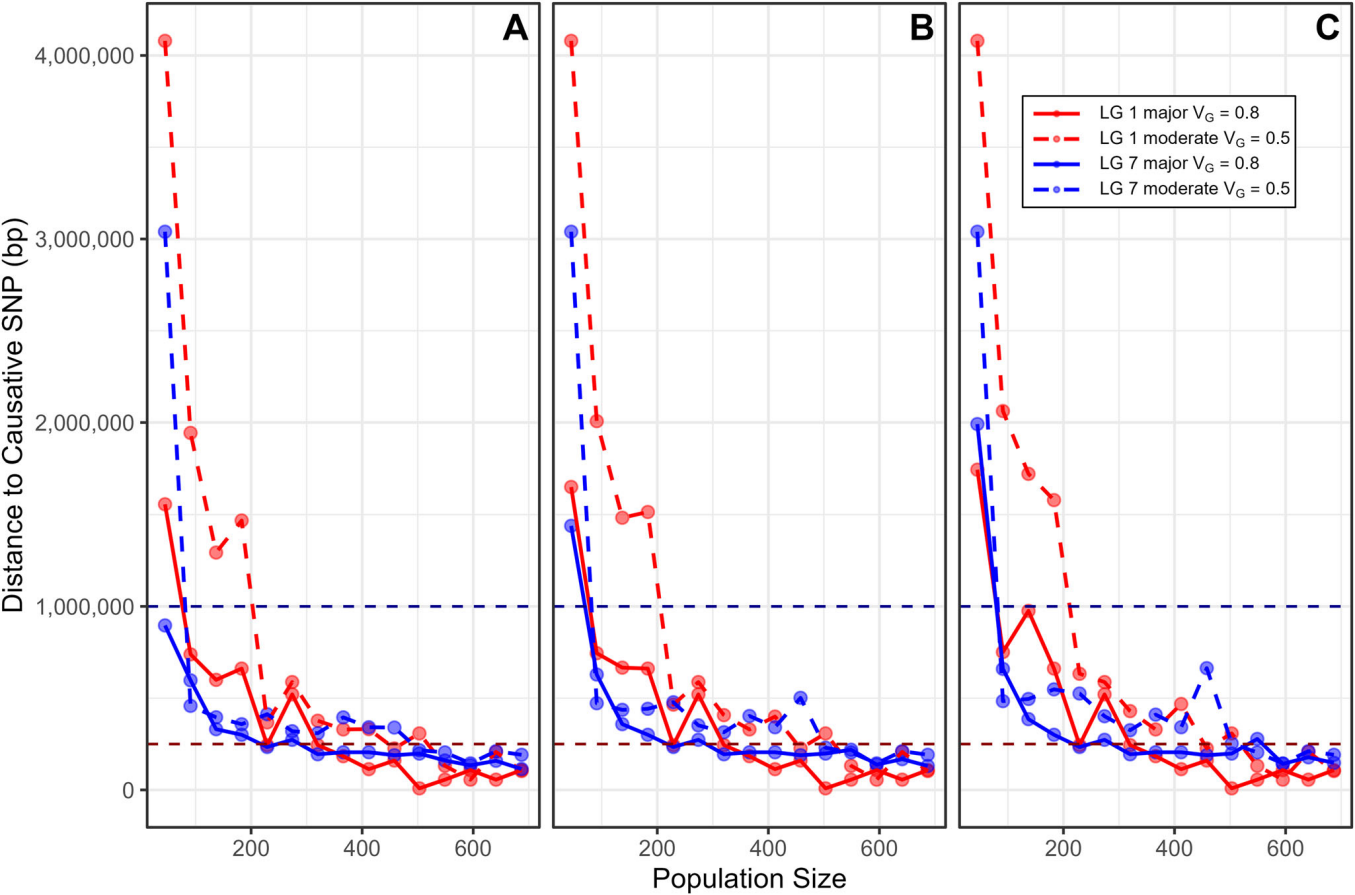
Resolution of marker–trait associations relative to the simulated quantitative trait locus (QTL) across population sizes. Distances were calculated using only significant single‐nucleotide polymorphisms (SNPs) located on the causal linkage group (LG). (A) Mean distance of closest significant SNP to simulated QTL. (B) Mean distance of the closest significant SNP to the simulated QTL. (C) Mean distance of the furthest significant SNP to the simulated QTL. The simulated QTL was located on either LG 1 (red) or LG 7 (blue) and evaluated under moderate genetic variance ($V_{G}$) = 0.5 (dashed lines) and major $V_{G}$ = 0.8 (solid lines). Each point represents the average resolution in base pairs across 40 simulation replicates at a given population size. Lower values indicate greater mapping precision. Horizontal dashed lines indicate 250 kb (red) and 1 Mb (blue), corresponding to the detection windows used in the power analysis and for evaluating fine‐mapping resolution.

**FIGURE 7:**
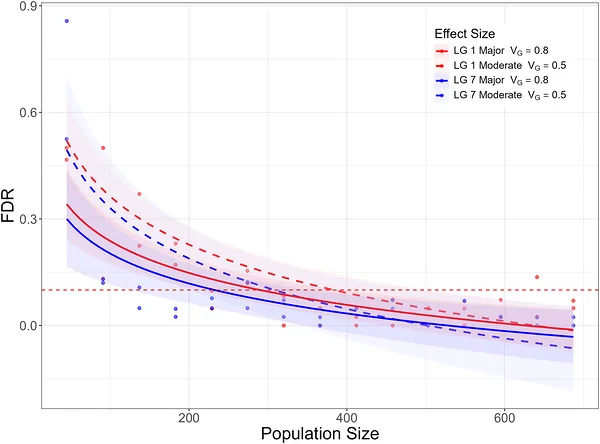
False discovery rate (FDR) displayed on the *y*‐axis for identifying marker–trait associations of a simulated quantitative trait locus (QTL) using a multi‐locus mixed model (MLMM). The QTL was simulated on two linkage groups (LGs) (LG 1 and LG 7) under two levels of genetic variance (0.5 = “moderate” and 0.8 = “major”) over 15 population sizes. Each point represents the average FDR at a given population size over 40 iterations of simulating the QTL. The dashed reference line at FDR = 0.1 highlights the threshold for acceptable false discovery using the MLMM. Scenario 7 is indicated by LG 1 major (red, solid), Scenario 6 is indicated by LG 1 moderate (red, dashed), Scenario 5 is indicated by LG 7 major (blue, solid), and Scenario 4 is indicated by LG 7 moderate (blue, dashed). Scenario 7 drops below FDR = 0.1 at *n* = 229, while Scenario 6 falls below 0.1 at *n* = 329. Scenario 5 drops below FDR = 0.1 at *n* = 137, and Scenario 4 crosses FDR = 0.1 at *n* = 183, reflecting that the simulated QTL on LG 7 falls under the FDR threshold of 0.1 at smaller population sizes. Additionally, larger sample sizes systematically reduce FDR across all LGs and effect sizes.

The power analysis showed that the ability to detect simulated QTLs varies with genetic variance explained, chromosomal location, and population size. Notably, the simulated QTL on LG 7 required smaller populations than the simulated QTL on LG 1 to reach the same power, suggesting that LD influences marker–trait associations. Published linkage maps of apple have shown that recombination rates can vary significantly among LGs, thus impacting the extent of LD decay (Di Pierro et al., 2016). For major effect QTLs, $V_{G}=0.8$, the statistical power reached the threshold of 0.8 at smaller population sizes compared to moderate effect QTLs, $V_{G}=0.5$. Across all simulation scenarios, increasing population size reduced the distance between marker–trait associations and the simulated QTL, indicating improved resolution (Figure 6; Tables S2–S4). At population sizes required to achieve a statistical power of 0.8 within the 500 kb window, the major effect QTL on LG 1 was localized to 661 kb from the simulated QTL across all three resolution metrics at *n* = 183, while the moderate effect QTL was localized to 377, 408, and 429 kb for the closest, average, and furthest distance metrics, respectively, at *n* = 320. For LG 7, the major effect QTL reached the power threshold within the 500 kb window at *n* = 320, with all three resolution metrics converging at 194 kb from the simulated QTL. Within the 2 Mb window, the 0.8 power threshold was reached at smaller population sizes, particularly for the LG 7 simulations, where the major effect QTL achieved closest, average, and furthest distances of 597, 629, and 659 kb, respectively, at *n* = 91. The FDR of the simulated QTL varies according to population size, LG (LG 1 vs. LG 7), and QTL effect size (moderate vs. major) (Figure 7, Table S5). We conducted an FDR analysis to measure the proportion of false positives among the significant SNPs in each scenario (Table 1). Overall, larger sample sizes reduce the FDR across all LGs and effect sizes. For instance, Scenario 6 (LG 1 moderate) remains above 0.1 at smaller populations but drops below FDR = 0.1 around *n* = 229 (FDR ≈ 0.098). By contrast, Scenario 7 first dips under 0.1 by *n* = 229 yet exhibits some variability around that population size (e.g., FDR ≈ 0.17 at *n* = 183 and decreasing to 0.048 by *n* = 229). Additionally, Scenario 5 crosses the 0.1 threshold at around *n* = 183 (FDR ≈ 0.024), underscoring its quicker drop in false discoveries compared to the LG 1 moderate scenario. These results confirm that major effect QTLs ($V_{G}$​ = 0.8) can meet an acceptable FDR threshold at smaller population sizes, whereas moderate effect QTLs ($V_{G}$​ = 0.5) require more individuals to achieve the same level of stringency. Across all simulation scenarios, increasing population size reduced the distance between the simulated QTL and both the closest and furthest associated SNPs, indicating progressive narrowing of the candidate interval surrounding the simulated locus (Figure 6; Tables S2–S4). Comparison of the 500 kb and 2 Mb detection windows showed that at populations sizes <250 all simulation scenarios consistently identified associations within the 2 MB region. Larger population sizes >500 were required for more fine mapping in the 500 kb region for all scenarios, with large‐effect QTLs reaching this threshold at smaller population sizes. The differences observed between the 500 kb and 2 Mb window sizes for the simulated QTL located in the low‐LD region on LG 1 showed that significant SNPs tended to occur either within the 500 kb window or outside the 2Mb window, with relatively few associations occurring at intermediate distances. Examination of the distribution of significant marker–trait associations (Figure S2) shows that simulation replicates that successfully detected the QTL within the broader 2 Mb window were also within the narrower 500 kb interval, producing similar power estimates across the two thresholds for several population sizes, but with further average distances to the simulated QTL due to the SNPs found outside the 2 Mb window.

## 4 DISCUSSION

We developed an OP‐based genetic mapping framework and successfully identified simulated QTL using the genetic variation present in a half‐sib F1 apple population. This simulation design represents a controlled baseline scenario in which the maternal parent contributes the segregating QTL. As such, the reported results should be interpreted as a proof‐of‐concept assessment rather than a model representing the full genetic complexity of other possible OP populations. Although a single major effect QTL was explicitly simulated, phenotypic variation also included polygenic background effects. Additional genetic complexity arising from paternal QTL contributions, multiple segregating loci, and other sources of genetic variation could influence mapping performance. These factors would likely reduce statistical power, decrease mapping resolution, and increase FDRs relative to the baseline estimates presented here. Future studies incorporating multiple segregating QTL, more complex genetic architectures, and empirical validation in diversity orchards will be necessary to fully evaluate the robustness and general applicability of the OP mapping framework. However, in species with long generation intervals and QTL donors with poor quality performance, mapping efforts will focus on traits with large to moderate effects as simulated in this study.

The results demonstrate the MLMM outperformed a standard MLM in detecting the simulated QTL. Using the MLM the simulated QTL on LG 3 was identified, but several false positives were observed across eight LGs: 1, 8, 9, 10, 11, 13, 14, and 15. MLM can yield spurious associations when background genetic effects are insufficiently accounted for, resulting in false positives that obscure the true marker–trait relationships. This has been seen in structured populations of *Arabidopsis thaliana*, apples, and humans (Atwell et al., 2010; Migicovsky et al., 2016; Platt et al., 2010). The marker–trait associations using the MLMM performed better than the MLM due to its ability to better account for multiple associated markers by including multiple loci through a stepwise approach (Segura et al., 2012). Populations with complex genetic structures can lead to spurious marker–trait associations (Newman et al., 2001), like in OP apple populations. While MLMs are effective at correcting for population structure by modeling it as a random effect (Kang et al., 2010; Yu et al., 2006). MLM tests one marker at a time and can miss the combined effects of multiple loci with small individual contributions. The MLMM addresses this limitation by allowing for the joint identification of multiple loci influencing the trait, enhancing the power to detect maker–trait associations while combining the advantages of MLM in correcting for population structure with the ability to model multiple effects jointly (Segura et al., 2012). Therefore, MLMM provided a more accurate mapping of the simulated QTL in our study and identified a significant marker–trait association in all scenarios (2–7) utilizing the MLMM.

The GBS dataset presented challenges due to improper variant calls in some of the F1 progeny in the populations. Approximately 8% of the sites had allelic states that were not possible in the biparental population when individuals’ allelic states were considered with respect to their parentage. BEAGLE allowed to impute agnostically to the parentage; however, the determination of impossible allelic states of some progeny could only be done because the parentage of the seven biparental populations were known. In an OP population that has unknown pollen donors, the ability to discover incorrect allelic states within the resulting mapping population would require additional analysis, potentially employing the use of principal component analysis or haplotype tracking (Hofmeister et al., 2022). Because imputation was performed without identifying parentage, imputation error would be expected to weaken true marker–trait associations, reducing statistical power and fine‐mapping resolution rather than creating false positives. As a result, the reported mapping performance and population size estimates represent lower bound expectations for lower coverage GBS datasets. Sequencing depth in different genotyping platforms poses another potential issue for genetic mapping in OP populations. Studies utilizing low‐depth sequencing data require careful consideration of structure, relatedness, and parentage for accurate imputation (Chan et al., 2016; Wang et al., 2024). A framework to reduce the resources needed would require efficient methods for accurate imputation if lower depth sequencing were to be used in F1 OP genetic mapping populations. Our results also show the importance of balancing population size and LD landscape to accurately map QTL with a range of effect sizes. Genetic mapping of a QTL on LG 1 with an average LD of 0.03 in a 25 kb region around the QTL showed a convergence with the power threshold at a population size of 183 F1s compared to the moderate effect QTL requiring a population size of 320 F1 individuals to converge with the power threshold. Mapping a QTL on LG 7 with an LD block with an average LD of 0.3 in a 2 kb region around the QTL showed a convergence with the power threshold at a population size of 137 individuals. The moderate effect QTL required a population size of 274 individuals to converge with the power threshold.

The difference in population sizes required to precisely map the QTL within the population could be due to the differing levels of genetic variation explained by the QTL, where lower effect QTL require greater mapping populations than larger effect QTL to accurately detect (Ball, 2005). This is due to the fact that QTL explaining less variation in the phenotype will have phenotypic variation from other sources, like environmental conditions and background genetic effects. In contrast, QTLs that explain more genetic variance can be detected with smaller mapping populations, as the basis for phenotypic variation is due to the QTL, and much less by environmental and other polygenetic effects, generating a stronger signal in marker–trait associations. The difference in population sizes required to identify marker–trait associations in contrasting strengths of LD blocks is likely due to the degree of linkage between the QTL and markers. LD, mapping population size, and QTL effect size were also important in the resolution of marker–trait associations with the simulated QTL.

Across the 40 repeated QTL simulations, increasing population size reduced the distance between significant markers and the simulated causal QTL across all resolution metrics of closest associated marker, mean of all associated markers, and furthest marker of each simulation iteration. Resolution improved more rapidly for the QTL simulated in the higher LD region on LG 7, reflecting the greater power to detect marker–trait associations at smaller population sizes. However, differences in fine‐mapping resolution occur not only from LD but also from variation in local recombination frequency across chromosomes, which can create genomic hotspots for recombination and thereby influence QTL resolution (Myers et al., 2005).

Comparison of the 2 Mb and 500 kb detection windows showed a larger impact on the higher LD region (LG 7). Within the broader 2 Mb window, the major effect QTL on LG 1 first exceeded the statistical power threshold of 0.8 at *n* = 183, whereas the moderate effect QTL first exceeded the threshold at *n* = 229. On LG 7, the major effect and moderate effect QTL first exceeded the threshold at *n* = 91. In contrast, under the more stringent 500 kb detection window, the major effect QTL on LG 1 first exceeded the threshold at *n* = 183, whereas the moderate effect QTL first exceeded the threshold at *n* = 229. The major effect QTL on LG 7 first exceeded the threshold at *n* = 320, while the moderate effect QTL did not consistently exceed the threshold until *n* = 595. These results indicate that higher LD regions facilitate QTL detection at smaller population sizes, whereas localization of associations within 500 kb of the causal locus requires substantially larger populations, particularly for moderate effect QTL. These results demonstrate that the OP‐based mapping framework is capable not only of detecting QTL but also of resolving associations to intervals sufficiently narrow to support downstream candidate gene identification and fine‐mapping applications.

Markers in higher LD with the QTL have greater marker–trait associations (Flint‐Garcia et al., 2003). However, QTL with tightly linked markers are more difficult to fine‐map and require greater population sizes to distinguish between markers tightly linked with the QTL (Y. Xu et al., 2017). The relationship between power and resolution observed in the low‐LD region demonstrates that these two measures capture different aspects of mapping performance. While resolution was influenced by a subset of more distant significant associations on LG 1, the distribution of detected markers indicates that many associations remained within the 500 kb window (Figure S2). Therefore, average resolution values alone may underestimate the practical utility of OP mapping for identifying closely linked markers in regions of low LD. This is particularly relevant for marker development, where the presence of tightly linked markers may be more important than the average distance of chromosome‐wide associations.

The analysis of the FDR for the simulations underscores the need to balance sample size with QTL effect size to control the proportion of false positives (Figure 7). Controlling FDR is essential for reliable marker–trait association studies (Storey & Tibshirani, 2003). We found that moderate effect QTLs exhibit higher FDRs at smaller population sizes, whereas major effect QTLs can achieve acceptable FDR thresholds more quickly. This pattern emphasizes that both effect magnitude and population size are important factors to consider for minimizing spurious associations, complementing the improvements in accuracy already provided by MLMM.

Paternal parent inference remains an issue with the proposed method of OP‐based genetic mapping, particularly when using sequence‐based genotyping. A two‐stage approach focusing on robust genotyping of potential pollen donors and imputing based off a subset, likely to be contributing to the OP F1 population, could improve accuracy despite having unknown paternal lines. In outcrossed, highly heterozygous species like apple, low sequencing depth and incomplete pedigree information can lead to ambiguous allele calls, thereby increasing the likelihood of imputation errors (Chan et al., 2016). The OP simulation analysis is done in a way that is agnostic to the pollen parents’ identity of the OP genetic mapping population, but it does assume accurate genotypic information and knowledge of parental QTL donors. As such, these simulations represent a best‐case scenario in which the pollen donors can be confirmed to have not contributed a large‐effect QTL to the trait. In practice, this would require screening of trees in the orchard to identify a mother tree that is resistant, while others remain susceptible. Future studies will focus on validating this approach for detection of sources of fireblight and apple scab resistance in the *M. sieversii* diversity orchard in Geneva, NY. Information on susceptibility of potential pollen donors will be used when selecting a resistant female parent. For breeding purposes, major resistance genes would be a prime candidate for this method as they explain much of the phenotypic variation and are capable of being screened early, at the seedling stage.

OP‐based genetic mapping is an intermediate method between biparental QTL mapping and GWAS. Like GWAS, OP populations contain heterogeneous relatedness and variable LD that are not a product of biparental crossing. In contrast to GWAS panels, OP progeny share a common maternal parent, introducing partial family structure. Although large diversity orchards are logistically demanding to establish, the OP framework described here is intended for use within existing, actively maintained orchards, where tree maintenance and land requirements are independent of OP progeny generation. Under these conditions, OP‐based genetic mapping reduces the additional logistical burden associated with controlled pollination, clonal propagation, and the establishment of dedicated biparental mapping populations. This allows OP‐based genetic mapping to leverage greater genetic diversity than biparental populations while still supporting QTL detection in the F1 OP population.

In a real OP population, a major consideration for which paternal inference would be necessary for accurate QTL identification is if there are any genetic effects contributed by one or more pollen donors that influence the genetic mapping through additivity or epistasis or contribute significantly to the phenotypic variation (Dong et al., 2024). However, the simulations indicate that it would be feasible to accurately map QTL within an OP population if there are no additional QTL contributing to the trait of interest segregating from the pollen donors, particularly for large‐effect QTL. In addition, residual genotype uncertainty arising from missing or inconsistent marker calls may further reduce mapping resolution in OP populations. Although direct imputation accuracy metrics were not evaluated in the present proof‐of‐concept study, imputation error would be expected to weaken true marker–trait associations and reduce statistical power rather than inflate false positive signals. As such, the simulation results presented here should be interpreted as conservative estimates of genetic mapping performance.

Fine mapping a QTL in an OP population can be particularly challenging. Not only are the paternal genotypes unknown, but the genomic location of the QTL may also be entirely undefined prior to mapping, complicating efforts to identify the precise region of interest. If a QTL falls within a region of high LD or low recombination, the ability to fine map in an F1 population could require restrictively large population sizes (Flint‐Garcia et al., 2003). However, fine mapping is done as a way to further explore genetic regions and identify potential candidate genes and variants for QTL that have already been mapped (Fahrentrapp et al., 2013; Soriano et al., 2014), and these considerations should be taken into account when selecting the maternal parent for OP genetic mapping. A two‐stage OP mapping framework could be utilized in which a smaller population is first used to identify the QTL region, followed by mapping with additional F1s based on the local recombination rate in the identified region. Genetic mapping populations of OP F1 apple could be used as a way to more efficiently generate fine mapping populations after a large potential region of a QTL has been identified. In the case where the LD in the QTL region is high, using an OP F1 genetic mapping population may not be feasible; however, lower LD regions or regions with higher recombination would allow fine mapping in an OP F1 population.

## 5 CONCLUSION

GWAS can be a useful QTL mapping method for apples that leverages the large amount of diversity found in core collections and germplasm repositories. However, the rapid LD decay, population genetic structure, and high heterozygosity in apples have restricted the widespread use of GWAS for QTL mapping in apples. Genetic mapping based on OP populations can accelerate the mapping of QTLs and development of reliable markers for MAS for woody perennial crops. Markers tightly linked to a QTL are especially important for reducing linkage drag and to avoid the risk of losing the desired trait due to recombination in MAS (Hospital, 2009). This underscores the necessity of precise, fine‐scale mapping to streamline the introgression of beneficial alleles while minimizing the inheritance of unwanted traits, particularly when using wild species. The ability to use OP populations for QTL mapping as an alternative to traditional QTL mapping approaches has the potential to significantly reduce the labor and costs associated with growing and maintaining biparental populations. The enhanced genetic diversity in the OP populations will also facilitate the detection of QTLs that are effective across a much wider range of genetic backgrounds. Although the simulations in this study were conducted solely in apples, further studies could extend the benefits of OP mapping to other perennial fruit tree crops.

GWAS has been valuable for QTL discovery in apples by leveraging the genetic diversity present in germplasm repositories and core collections. However, challenges such as rapid LD decay, high levels of population structure, and high heterozygosity limit its broader application in QTL mapping. In contrast, OP populations offer a practical alternative for genetic mapping in woody perennials. By utilizing natural pollination within genetically diverse orchards, OP mapping reduces the time, labor, and costs associated with maintaining large biparental populations.

Importantly, OP‐based mapping can accelerate the identification of tightly linked markers for MAS, which are critical for minimizing linkage drag and preserving desired traits when developing new varieties, especially when incorporating alleles from wild relatives (Hospital, 2009). The increased genetic diversity captured in OP populations may also improve the detection of QTLs effective across diverse genetic backgrounds. While this study focused on apples, the mapping framework developed here has broad potential. Future research could expand its application to other perennial fruit tree crops, offering a more efficient path to trait discovery and marker development in complex, heterozygous species.

## AUTHOR CONTRIBUTIONS

**David Hickok**: Formal analysis; investigation; methodology; visualization; writing—original draft. **Awais Khan**: Conceptualization; funding acquisition; supervision; writing—review and editing. **Kelly Robbins**: Conceptualization; funding acquisition; methodology; supervision; writing—review and editing.

## CONFLICT OF INTEREST STATEMENT

The authors declare no conflicts of interest.

## Supporting information



Supplementary Tables S1–S5 report statistical power, false discovery rate, and resolution metrics for all simulation scenarios across population sizes under both 500 kb and 2 Mb QTL detection windows. Resolution analyses include the mean closest significant SNP distance, mean significant SNP distance, and mean furthest significant SNP distance relative to the simulated causal QTL. Supplementary Figure 1 reports the distribution of simulated phenotype values across all repeated simulations and population sizes for LG1 and LG 7. Supplementary Figure 2 shows a violin plot of the density of significant SNPs detected on the same chromosome as the simulated QTL referenced with the window ranges used for calculating power.

## Data Availability

The genotype‐by‐sequencing SNP dataset for the populations GMAL4590, GMAL4591, GMAL4592, GMAL4593, GMAL4594, GMAL4595, and GMAL4596 used to calculate the genomic relationship matrix, simulate phenotypes, and perform marker‐trait associations is made available on the Dryad database (https://doi.org/10.5061/dryad.vhhmgqp5z). The link to simulations is available in GitHub at the following link: https://github.com/dh564/OP_Sim.
