# Supplementary material for: Development of an open‐pollinated genetic mapping framework to facilitate the identification of QTL in apples
Source: Plant Genome. 2026 Aug 16;19(3):e70288. doi: 10.1002/tpg2.70288 (PMC13492597; doi:10.1002/tpg2.70288)
Supplement: Supplementary file 1 [file TPG2-19-e70288-s001.docx]

**Supplementary Material for:**

**Development of an open-pollinated genetic mapping framework to facilitate the identification of QTL in apples**

David Hickok^1, 2^, Awais Khan^1^, Kelly Robbins^2^

^1^Plant Pathology and Plant-Microbe Biology Section, School of Integrative Plant Science, Cornell University, Geneva, NY, 14456, USA

^2^Plant Breeding and Genetics Section, School of Integrative Plant Science, Cornell University, Ithaca, NY, 14853, USA

Number of pages: 9

Number of Tables: 5

Number of Figures: 2

# SUPPLEMENTAL DATA

Supplemental Table 1. Average power to detect the simulated QTL across the 40 repeated simulations at each population size within a 500kb and 2Mb window of the QTL for both genetic variances ($\boldsymbol{V}_{\boldsymbol{G}}$) of 0.5 and 0.8

| **Average Power LG1** | | | | | **Average Power LG7** | | | | |
| --- | --- | --- | --- | --- | --- | --- | --- | --- | --- |
|  | $\boldsymbol{V}_{\boldsymbol{G}}$ **= 0.8** | | $\boldsymbol{V}_{\boldsymbol{G}}$ **= 0.5** | |  | $\boldsymbol{V}_{\boldsymbol{G}}$**= 0.8** | | $\boldsymbol{V}_{\boldsymbol{G}}$ **= 0.5** | |
| **Window** | **500kb** | **2Mb** | **500kb** | **2Mb** | **Window** | **500kb** | **2Mb** | **500kb** | **2Mb** |
| **n** |  |  |  |  | **n** |  |  |  |  |
| 45 | 0.40 | 0.40 | 0.00 | 0.00 | 45 | 0.44 | 0.67 | 0.40 | 0.40 |
| 91 | 0.58 | 0.58 | 0.27 | 0.27 | 91 | 0.54 | 0.83 | 0.69 | 0.81 |
| 137 | 0.71 | 0.71 | 0.60 | 0.60 | 137 | 0.69 | 0.90 | 0.53 | 0.87 |
| 183 | 0.85 | 0.85 | 0.41 | 0.41 | 183 | 0.69 | 0.95 | 0.68 | 0.87 |
| 229 | 0.88 | 0.88 | 0.81 | 0.81 | 229 | 0.75 | 0.95 | 0.53 | 0.91 |
| 274 | 0.73 | 0.73 | 0.70 | 0.70 | 274 | 0.70 | 0.98 | 0.69 | 0.87 |
| 320 | 0.88 | 0.88 | 0.83 | 0.83 | 320 | 0.80 | 1.00 | 0.59 | 0.95 |
| 366 | 0.90 | 0.90 | 0.83 | 0.83 | 366 | 0.78 | 0.98 | 0.58 | 0.93 |
| 412 | 0.93 | 0.93 | 0.80 | 0.80 | 412 | 0.73 | 1.00 | 0.68 | 0.92 |
| 458 | 0.93 | 0.93 | 0.88 | 0.88 | 458 | 0.75 | 1.00 | 0.60 | 0.95 |
| 503 | 1.00 | 1.00 | 0.85 | 0.85 | 503 | 0.78 | 1.00 | 0.70 | 1.00 |
| 549 | 0.98 | 0.98 | 0.93 | 0.93 | 549 | 0.83 | 1.00 | 0.78 | 0.98 |
| 595 | 0.95 | 0.95 | 0.98 | 0.98 | 595 | 0.88 | 1.00 | 0.80 | 1.00 |
| 641 | 0.98 | 0.98 | 0.90 | 0.90 | 641 | 0.83 | 1.00 | 0.80 | 0.98 |
| 687 | 0.95 | 0.95 | 0.95 | 0.95 | 687 | 0.88 | 1.00 | 0.75 | 1.00 |

Supplemental Table 2. Distance to the simulated QTL in base pairs. The average distance to the causative marker is reported across the closest significant marker for each of the 40 repeated simulations at each population size for both genetic variances ($\boldsymbol{V}_{\boldsymbol{G}}$) of 0.5 and 0.8.

| **Average Closest Distance to QTL** | | | | | |
| --- | --- | --- | --- | --- | --- |
| **Fine Mapping Resolution (bp) on LG 1** | | | **Fine Mapping Resolution (bp) on LG 7** | | |
| **n** | $\boldsymbol{V}_{\boldsymbol{G}}$ **= 0.8** | $\boldsymbol{V}_{\boldsymbol{G}}$ **= 0.5** | **n** | $\boldsymbol{V}_{\boldsymbol{G}}$ **= 0.8** | $\boldsymbol{V}_{\boldsymbol{G}}$ **= 0.5** |
| 45 | 1.56E+06 | 4.08E+06 | 45 | 8.96E+05 | 3.04E+06 |
| 91 | 7.39E+05 | 1.94E+06 | 91 | 5.97E+05 | 4.58E+05 |
| 137 | 6.00E+05 | 1.29E+06 | 137 | 3.31E+05 | 3.96E+05 |
| 183 | 6.61E+05 | 1.47E+06 | 183 | 3.01E+05 | 3.58E+05 |
| 229 | 2.44E+05 | 3.68E+05 | 229 | 2.34E+05 | 4.13E+05 |
| 274 | 5.20E+05 | 5.88E+05 | 274 | 2.73E+05 | 3.20E+05 |
| 320 | 2.44E+05 | 3.77E+05 | 320 | 1.94E+05 | 3.09E+05 |
| 366 | 1.84E+05 | 3.30E+05 | 366 | 2.05E+05 | 3.95E+05 |
| 412 | 1.13E+05 | 3.31E+05 | 412 | 2.05E+05 | 3.42E+05 |
| 458 | 1.60E+05 | 2.26E+05 | 458 | 1.89E+05 | 3.40E+05 |
| 503 | 8.60E+03 | 3.08E+05 | 503 | 1.98E+05 | 2.16E+05 |
| 549 | 5.59E+04 | 1.32E+05 | 549 | 1.60E+05 | 2.04E+05 |
| 595 | 1.08E+05 | 5.59E+04 | 595 | 1.34E+05 | 1.45E+05 |
| 641 | 5.59E+04 | 2.07E+05 | 641 | 1.59E+05 | 2.11E+05 |
| 687 | 1.08E+05 | 1.03E+05 | 687 | 1.14E+05 | 1.92E+05 |

Supplemental Table 3. Distance to the simulated QTL in base pairs. The average distance to the causative marker is reported across all significant markers for each of the 40 repeated simulations at each population size for both genetic variances ($\boldsymbol{V}_{\boldsymbol{G}}$) of 0.5 and 0.8.

| **Average Distance to QTL** | | | | | |
| --- | --- | --- | --- | --- | --- |
| **Fine Mapping Resolution (bp) on LG 1** | | | **Fine Mapping Resolution (bp) on LG 7** | | |
| **n** | $\boldsymbol{V}_{\boldsymbol{G}}$ **= 0.8** | $\boldsymbol{V}_{\boldsymbol{G}}$ **= 0.5** | **n** | $\boldsymbol{V}_{\boldsymbol{G}}$ **= 0.8** | $\boldsymbol{V}_{\boldsymbol{G}}$ **= 0.5** |
| 45 | 1.65E+06 | 4.08E+06 | 45 | 1.44E+06 | 3.04E+06 |
| 91 | 7.45E+05 | 2.01E+06 | 91 | 6.29E+05 | 4.72E+05 |
| 137 | 6.66E+05 | 1.48E+06 | 137 | 3.58E+05 | 4.36E+05 |
| 183 | 6.61E+05 | 1.51E+06 | 183 | 3.01E+05 | 4.42E+05 |
| 229 | 2.44E+05 | 4.65E+05 | 229 | 2.34E+05 | 4.78E+05 |
| 274 | 5.20E+05 | 5.88E+05 | 274 | 2.73E+05 | 3.52E+05 |
| 320 | 2.44E+05 | 4.08E+05 | 320 | 1.94E+05 | 3.14E+05 |
| 366 | 1.84E+05 | 3.30E+05 | 366 | 2.05E+05 | 4.03E+05 |
| 412 | 1.13E+05 | 4.00E+05 | 412 | 2.05E+05 | 3.42E+05 |
| 458 | 1.60E+05 | 2.26E+05 | 458 | 1.89E+05 | 5.02E+05 |
| 503 | 8.60E+03 | 3.08E+05 | 503 | 1.98E+05 | 2.30E+05 |
| 549 | 5.59E+04 | 1.32E+05 | 549 | 2.19E+05 | 2.04E+05 |
| 595 | 1.08E+05 | 5.59E+04 | 595 | 1.38E+05 | 1.45E+05 |
| 641 | 5.59E+04 | 2.07E+05 | 641 | 1.68E+05 | 2.11E+05 |
| 687 | 1.08E+05 | 1.03E+05 | 687 | 1.31E+05 | 1.92E+05 |

Supplemental Table 4. Distance to the simulated QTL in base pairs. The average distance to the causative marker is reported across the furthest significant marker for each of the 40 repeated simulations at each population size for both genetic variances ($\boldsymbol{V}_{\boldsymbol{G}}$) of 0.5 and 0.8.

| **Average Furthest Distance to QTL** | | | | | |
| --- | --- | --- | --- | --- | --- |
| **Fine Mapping Resolution (bp) on LG 1** | | | **Fine Mapping Resolution (bp) on LG 7** | | |
| **n** | $\boldsymbol{V}_{\boldsymbol{G}}$ **= 0.8** | $\boldsymbol{V}_{\boldsymbol{G}}$ **= 0.5** | **n** | $\boldsymbol{V}_{\boldsymbol{G}}$ **= 0.8** | $\boldsymbol{V}_{\boldsymbol{G}}$ **= 0.5** |
| 45 | 1.74E+06 | 4.08E+06 | 45 | 1.99E+06 | 3.04E+06 |
| 91 | 7.51E+05 | 2.06E+06 | 91 | 6.59E+05 | 4.84E+05 |
| 137 | 9.75E+05 | 1.72E+06 | 137 | 3.86E+05 | 4.96E+05 |
| 183 | 6.61E+05 | 1.58E+06 | 183 | 3.01E+05 | 5.48E+05 |
| 229 | 2.44E+05 | 6.33E+05 | 229 | 2.34E+05 | 5.25E+05 |
| 274 | 5.20E+05 | 5.88E+05 | 274 | 2.73E+05 | 4.02E+05 |
| 320 | 2.44E+05 | 4.29E+05 | 320 | 1.94E+05 | 3.25E+05 |
| 366 | 1.84E+05 | 3.30E+05 | 366 | 2.05E+05 | 4.10E+05 |
| 412 | 1.13E+05 | 4.68E+05 | 412 | 2.05E+05 | 3.42E+05 |
| 458 | 1.60E+05 | 2.26E+05 | 458 | 1.89E+05 | 6.64E+05 |
| 503 | 8.60E+03 | 3.08E+05 | 503 | 1.98E+05 | 2.53E+05 |
| 549 | 5.59E+04 | 1.32E+05 | 549 | 2.78E+05 | 2.04E+05 |
| 595 | 1.08E+05 | 5.59E+04 | 595 | 1.42E+05 | 1.45E+05 |
| 641 | 5.59E+04 | 2.07E+05 | 641 | 1.78E+05 | 2.11E+05 |
| 687 | 1.08E+05 | 1.03E+05 | 687 | 1.47E+05 | 1.92E+05 |

Supplemental Table 5. False discovery rate (FDR) at each population size based on the 40 repeated simulations across the 15 different population sizes for both genetic variances ($\boldsymbol{V}_{\boldsymbol{G}}$) of 0.5 and 0.8. Top of FormBottom of Form

| **FDR on LG1** | | | **FDR on LG7** | | |
| --- | --- | --- | --- | --- | --- |
| **n** | $V_{G}$**= 0.8** | $V_{G}$**= 0.5** | **n** | $V_{G}$ **= 0.8** | $V_{G}$**= 0.5** |
| 45 | 0.47 | 0.50 | 45 | 0.53 | 0.86 |
| 91 | 0.13 | 0.50 | 91 | 0.12 | 0.13 |
| 137 | 0.22 | 0.37 | 137 | 0.05 | 0.11 |
| 183 | 0.17 | 0.23 | 183 | 0.02 | 0.05 |
| 229 | 0.05 | 0.10 | 229 | 0.05 | 0.08 |
| 274 | 0.12 | 0.15 | 274 | 0.05 | 0.12 |
| 320 | 0.00 | 0.08 | 320 | 0.02 | 0.07 |
| 366 | 0.05 | 0.00 | 366 | 0.00 | 0.02 |
| 412 | 0.00 | 0.02 | 412 | 0.05 | 0.05 |
| 458 | 0.07 | 0.00 | 458 | 0.05 | 0.07 |
| 503 | 0.05 | 0.05 | 503 | 0.00 | 0.00 |
| 549 | 0.00 | 0.05 | 549 | 0.07 | 0.07 |
| 595 | 0.07 | 0.02 | 595 | 0.02 | 0.02 |
| 641 | 0.02 | 0.14 | 641 | 0.02 | 0.00 |
| 687 | 0.05 | 0.07 | 687 | 0.02 | 0.00 |

Supplemental Figure 1. Phenotypic distribution for simulation scenarios 4-7 across all population sizes. The simulation scenario is indicated by the column labels and the population sizes are indicated by the row labels.

Supplemental Figure 2. Violin plots show the distribution of base pair (bp) distances between significant SNPs and the simulated QTL across 40 replicated simulations at each population size for both genetic variances ($\boldsymbol{V}_{\boldsymbol{G}}$) of 0.5 and 0.8. Dashed horizontal lines indicate distances of 250 kb (red) and 1 Mb (blue) from the causal QTL.
